# Monthly Summary

**Published:** 1880-04

**Authors:** 


### MONTHLY SUMMARY.

*Chloral as an Anæsthetic for Children.*—M. Redier, in *Jour. des Sciences Medic. de Lille*, says that chloral, in sufficiently large doses, always produces in children a profound sleep approaching to anæsthesia.

In incomplete anæsthesia from chloroform, the patient feels the pain, and manifests his sensations by cries, as in anæsthesia from chloral; but, while in the first case memory is preserved, in the latter all recollection of what was done is lost, which is of advantage in the case of children.

Chloral given in large doses to the latter (2–4 grammes) gives rise to no trouble. In the only fatal case recorded, 5 grammes was given to a child less than three years old. The necessary doses for anæsthesia are: From 2 to 4 years, 2 grammes; from 4 to 8, 3 grammes; from 8 to 12, 4 grammes. It should be given in one does, while fasting.

Sleep comes on very quickly. The operation should be performed about an hour or an hour and a half after the child has fallen to sleep. Five hours is the usual duration of the sleep, during which the respiration and the circulation preserve very nearly the normal type.

This method of anæsthesia can be advantageously employed for the extraction of teeth, destruction of nævi, application of caustics, opening of abscesses, and for long and painful dressings, as of extensive burns.—*Druggists' Circular.*

---

*Death from Chloroform in a Dentist's Chair.*—It is our painful duty to record another death from chloroform in a dentist's chair. The patient was a young man, 21 years of age, and the place where the accident occurred was St. Johnsbury, Vt. No details are given—simply that death was the result of the administration of chloroform. To most physicians such a statement is enough to bring to mind the usual relations of cause and effect in these cases, and to emphasize a growing conviction that this dangerous anæsthetic should be banished from the dental operating-room. In view of the number of deaths from chloroform during dental operations, and the usual distressing circumstances which attend them, it is indeed surprising that any dentist should, even under the most extraordinary circumstances, run any risks with his patient. We are not prepared to say that the dentist did not use all the ordinary precautions in inducing anæsthesia. Probably he did. But he used treacherous chloroform as the agent, and for this he is certainly in the highest degree culpable.—*Med. Record.*

---

*The Effects of Tobacco.*—Dr. C. R. Drysdale writes:

My own experience of the evil effects of great tobacco smoking and chewing, is that these are among the most prevalent causes of chronic disease in the male sex. Of course, I do not mean for one moment to compare the dangers caused by the use of tobacco with those we are so familiar with at the bedside, in cases of diseases caused by alcohol. Tobacco does not cause cirrhosis of the liver, nor disease of the lungs and heart, in the same way, or with the same frequency as chronic tippling does. But there are, nevertheless, several well marked diseases caused by the taking in of nicotine into the blood, whether through the absorbents of the mouth in smoking, or, more rapidly, in the case of chewing. First of all, the digestive organs are often greatly impaired by the use of nicotianæ tabacum. The teeth are frequently blackened, and the gums swollen in great smokers and chewers. Caries of the teeth is favored by the various acids produced by the burning of tobacco, and mingled with the saliva. Duskiness of the fauces, and relaxed sore throat, are far too prevalent among smokers, as good observers have long noticed. Dyspepsia, caused by nicotine, is so common as to be hardly worth referring to. Diarrhœa, or more frequently, constipation, is induced by the use of tobacco in many instances. And I must not omit, in passing, the remark that the *male sex* who smoke are alone, with the very rarest exceptions, the subjects of epithelioma of the lip. I once saw such a case in an old Irish woman, who was a constant pipe smoker.

With regard to the nervous system, the weakness of vision produced by nicotine is a constant trouble to youthful smokers. Mr. George Critchett has remarked that among wealthy young men weak sight is very frequently caused by their extravagant addiction to cigars and pipes. Tobacco amaurosis, too, is far from rare. In very young men, the use of nicotine is peculiarly inimical to intellectual improvement. Thus, M. Joly found in the Polytechnic School at Paris that the non-smoking students carried off the very great majority of the prizes for Mathematics; and Dr. Kostral, physician to the State factory of tobacco, of Austria, shows how nicotine poisoning often kills the young boys of the factory, and causes abortions in the young mothers, and death of infants at the breast, through the nicotine contained in their mother's milk.—*Med. Press and Circular.*

---

*Effects of Tea on the System.*—1. With tea, as with any potent drug, there is a proper and improper dose.

2.—In moderation, tea is a mental and bodily stimulant of a most agreeable nature, followed by no harmful reaction. It produces contentment of mind, allays hunger and bodily weariness, and increases the incentive and the capacity for work.

3.—Taken immoderately, it leads to a very serious group of symptoms, such as headache vertigo, heat and flushings of body, ringing in the ears, mental dullness and confusion, tremulousness, nervousness, sleeplessness, apprehension of evil, exhaustion of mind and body, with disinclination to mental and physical exertion, increased and irregular action of the heart, increased respiration.

Each of the above symptoms is produced by tea taken in immoderate quantities, irrespective of dyspepsia, or hypochondria, or hyperæmia. The prolonged use of tea produces, additionally, symptoms of these three latter diseases. In short, in immoderate doses, tea has a most injurious effect upon the nervous system.

4.—Immoderate tea drinking, continued for a considerable time, with great certainty produces dyspepsia.

5.—The immediate mental symptoms produced by tea are not to be attributed to dyspepsia.

In the above experiment upon myself, the whole group of symptoms was produced, with no sign of digestive trouble superadded.

6.—Tea retards the "waste," or retrograde metamorphosis of tissue, and thereby diminishes the demand for food.

It also diminishes the amount of urine secreted.

7.—Many of the symptoms of immoderate tea drinking are such as may occur without suspicion of tea being their cause; and we find many people taking tea to relieve the very symptoms which its abuse is producing.—*Dr. Morton, in Journal of Mental and Nervous Diseases.*

